# Optimizing the use of lenvatinib in combination with pembrolizumab in patients with advanced endometrial carcinoma

**DOI:** 10.3389/fonc.2022.979519

**Published:** 2022-09-21

**Authors:** Domenica Lorusso, Romano Danesi, Laura Deborah Locati, Gianluca Masi, Ugo De Giorgi, Angiolo Gadducci, Sandro Pignata, Roberto Sabbatini, Antonella Savarese, Giorgio Valabrega, Claudio Zamagni, Nicoletta Colombo

**Affiliations:** ^1^ Department of Clinical Research Planning, Fondazione Policlinico Universitario A Gemelli Istituto di Ricerca e Cura a carattere scientifico (IRCCS), Rome, Italy; ^2^ Department of Life Science and Public Health, Catholic University of Sacred Heart, Rome, Italy; ^3^ Unit of Clinical Pharmacology and Pharmacogenetics, Department of Clinical and Experimental Medicine, University of Pisa, Pisa, Italy; ^4^ Translational Oncology Unit, Istituto di Ricerca e Cura a carattere scientifico (IRCCS) Istituti Clinici Scientifici (ICS) Maugeri, Pavia, Italy; ^5^ Department of Internal Medicine and Medical Therapy, University of Pavia, Pavia, Italy; ^6^ Unit of Medical Oncology 2, Azienda Ospedaliero-Universitaria Pisana, Pisa, Italy; ^7^ Department of Translational Research and New Technologies in Medicine and Surgery, University of Pisa, Pisa, Italy; ^8^ Department of Medical Oncology, Istituto di Ricerca e Cura a carattere scientifico (IRCCS) Istituto Romagnolo per lo Studio dei Tumori (IRST), Dino Amadori, Meldola, Italy; ^9^ Department of Clinical and Experimental Medicine, Division of Gynecology and Obstetrics, University of Pisa, Pisa, Italy; ^10^ Department of Urology and Gynecology, Istituto Nazionale Tumori Istituto di Ricerca e Cura a carattere scientifico (IRCCS) “Fondazione Giovanni Pascale”, Naples, Italy; ^11^ Division of Medical Oncology 1, Istituto di Ricerca e Cura a carattere scientifico (IRCCS) -Regina Elena National Cancer Institute, Rome, Italy; ^12^ University of Torino-Struttura Complessa a Direzione Universitaria (S.C.D.U.) Oncologia Azienda Ospedaliera (A.O) Ordine Mauriziano-Ospedale Umberto I, Torino, Italy; ^13^ Addarii Medical Oncology, Istituto di Ricerca e Cura a carattere scientifico (IRCCS) Azienda Ospedaliero-Universitaria di Bologna, Bologna, Italy; ^14^ School of Medicine and Surgery, University of Milan-Bicocca, Milan, Italy; ^15^ Department of Oncological Gynecology, European Institute of Oncology (IEO) Istituto di Ricerca e Cura a carattere scientifico (IRCCS), Milan, Italy

**Keywords:** lenvatinib, pembrolizumab, endometrial cancer, tyrosine kinase inhibitor, immune response

## Abstract

**Introduction:**

The combination of lenvatinib plus pembrolizumab demonstrated a relevant clinical benefit in patients with endometrial carcinoma. The safety profile was consistent with the established profiles of each drug in monotherapy, with the most frequent adverse events being hypertension, an on-target effect, hypothyroidism, diarrhea, nausea, vomiting, loss of appetite, fatigue, and weight loss.

**Areas covered:**

We first review the rationale based on the combination of a VEGFR inhibitor and an immune checkpoint inhibitor, highlighting the main pharmacokinetic and pharmacodynamic features of lenvatinib. Next, we focus on the common adverse events associated with lenvatinib and guide how to optimally prevent, detect, and manage them, while minimizing interruptions during lenvatinib treatment.

**Discussion:**

The side effects profile of lenvatinib is very well known, being similar across different tumor types. Most toxicities can be preventable. An appropriate, proactive, and thorough management of lenvatinib toxicities during treatment is required to maximize potential lenvatinib efficacy. Adverse events should be detected as early as possible, by both carefully monitoring the patient from lenvatinib initiation and preventing their occurrence. Patients should be followed also during treatment as some adverse events, e.g., cardiac dysfunction might appear later. Increased awareness on risk to benefit ratio among clinicians would be helpful to avoid dose interruptions or discontinuation of lenvatinib, with preferring other medical interventions and supportive care.

## Introduction

Endometrial cancer is the most common gynecologic malignancy, with an estimated 65,950 new cases and 12,550 deaths in 2022 in the United States (1). Although endometrial carcinoma is a disease usually associated with older age, it can present in women at any age. Most endometrial carcinomas result from a spontaneous mutation, but up to 30% of cases are associated with germline mutations in mismatch repair genes (deficient mismatch repair dMMR) or show microsatellite instability-high (MSI-H) (2). Front-line treatments for women with advanced endometrial carcinoma are established as platinum-based chemotherapy plus taxane and hormone therapy, especially in women with low-grade endometrioid tumors and smaller tumor volume (3). Second-line treatments for recurrent patients have been an unmet clinical need until the recent approval of pembrolizumab for patients with MSI-H (4). For patients without MSI-H/deficient MMR (dMMR), the combination of lenvatinib plus pembrolizumab has emerged as a potential therapeutic opportunity (5, 6).

In this review, we summarize the main molecular, pharmacokinetic, and pharmacodynamic features of the combination of lenvatinib and pembrolizumab and focus on the management of lenvatinib in endometrial carcinoma based on the results from clinical trials and the experience acquired in other tumors.

## Role of VEGF in tumor immune editing and rationale for the combination of anti-VEGF and immunotherapy

Vascular Endothelial Growth Factor (VEGF) is a cytokine with dual action in tumor biology. On one hand, hypoxic cancer cells and vascular endothelial cells release VEGF that favors angiogenesis, tumor growth, invasion, and metastasis; on the other, VEGF induces the mobilization and proliferation of various cells, including regulatory T cells (Tregs), the release of immunosuppressive cytokines, thus leading to immune escape (7). The inhibition of VEGF receptors (VEGFR) with targeted drugs impacts immune response: dendritic cells show an increased antigen presentation, and T cells are activated in the priming phase and migrate from lymph nodes to tumor sites. In addition, anti-VEGFRs suppress the generation of Tregs, tumor-associated macrophages, and myeloid-derived suppressor cells at the tumor site and abrogate the expression of immunosuppressive cytokines such as TGF-β and IL-10. Therefore, these drugs reprogram the immunosuppressive tumor microenvironment into an immunostimulatory environment; under these conditions, immunotherapy with PD-1/PD-L1 antibodies further enhances the antitumor activity of T cells (7).

The combined antitumor activity of lenvatinib plus anti-PD-1 was investigated in animal models (CT-26 mice) (8). Treatment with lenvatinib or an anti-PD-1 alone significantly inhibited the *in vivo* tumor growth of CT26 isografts compared with the vehicle; however, the combination of lenvatinib plus anti-PD-1 drugs synergically suppressed tumor growth compared with either treatment alone. The activity was more evident in immune-competent animals than in their immunosuppressed counterparts (8). Therefore, the rationale to combine lenvatinib and pembrolizumab is based on the synergic effect that these drugs exert on the immune system.

## Pharmacodynamic and pharmacokinetics of lenvatinib

Lenvatinib is a tyrosine kinase inhibitor that selectively blocks VEGFR, PDGFR, RET, and cKIT (9), with a higher potency, especially against VEGFR-2 and VEGFR-3, than other tyrosine kinase inhibitors (TKI) such as cabozantinib, pazopanib, and sunitinib (10). Like other TKIs, lenvatinib binds the ATP binding pocket of kinases in its active conformation (9). The ATP binding site is a highly conserved domain between kinases, and this explains the relative lack of single kinase selectivity and the ability to act on multiple targets (9). This behavior may represent an advantage since lenvatinib can inhibit other receptors involved in angiogenesis.

Lenvatinib binds the ATP binding pocket in a peculiar mode. Indeed, kinetic studies revealed that lenvatinib had a rapid association rate constant and a relatively slow dissociation rate constant in complex with VEGFR2 and interacted with a region neighboring the kinase ATP-binding site of VEGFR2. This interaction may contribute to prolonging the binding time compared with that of other inhibitors, such as sorafenib (11).

*In vivo* data indicated that lenvatinib is extensively metabolized through non-P450-mediated pathways, including oxidation by aldehyde oxidase, glutathione conjugation with the elimination of the O-aryl group (chlorophenyl moiety), and combinations of these pathways followed by further biotransformation (e.g., glucuronidation, hydrolysis of the glutathione moiety, degradation of the cysteine moiety, and intramolecular rearrangement of the cysteinyl-glycine and cysteine conjugates with subsequent dimerization). In the liver, cytochrome P450 3A4 is the predominant isoform that metabolizes lenvatinib by methylation; however, hepatic metabolism is not relevant and, thus, *in vivo*, inducers and inhibitors of CYP 3A4 show a minimal effect on lenvatinib exposure. As expected from a low CYP 3A4 metabolism, no gender differences were observed in the PD and PK profile of lenvatinib, and no clinically relevant drug-drug interactions had been reported (12).

Lenvatinib half-life is of about 28 hours; therefore, it is enough to cover the entire period between administrations, but not so long as to ensure a reasonably short and fast clearance, in case of adverse reactions (10, 12).

## Dosing

Lenvatinib showed a high binding to human plasma proteins, especially alpha1-glycoprotein and gamma-globulin, which increases the apparent volume of distribution at a steady-state (12). In addition, the distribution of drugs in tumor angiogenesis is always very difficult, due to the structural abnormalities of blood vessels. At treatment initiation, it is advisable to use the full lenvatinib dose to quickly saturate the distribution volume and achieve an effective concentration at a steady state that guarantees antitumoral activity. On the contrary, a progressive increase in concentration may need a considerable number of days or even weeks to reach the effective concentration and exposes the patient to subtherapeutic concentrations, which are not able to block tumor vascularization. In animal models, sunitinib showed transient antitumor effects as well as dynamic changes in the VEGF pathway: it initially blocked tumor growth, but after drug discontinuation, the tumor rapidly regrew (13). Therefore, dose titration and the use of lenvatinib at a sub-optimal concentration to prevent adverse reactions may be detrimental.

A randomized study specifically investigated the efficacy and safety of lenvatinib 18 mg versus 24 mg to understand whether a lower dose of lenvatinib would provide comparable efficacy but improved safety relative to the approved 24-mg/day starting dose in patients with thyroid carcinoma. The study did not demonstrate noninferiority of lenvatinib 18 mg compared to the approved dose and the 17% difference in the overall response rate at 24 weeks and overall response rate indicated that lenvatinib 24 mg provides a clinically relevant higher activity than the reduced dose; the safety analysis, on the contrary, did not reveal any advantage in terms of adverse reactions incidence and the overall safety profile of two dosings was similar (14). Therefore, starting at the recommended dose, with dose reductions if required, is important for optimizing lenvatinib treatment.

In endometrial cancer the approved dose is 20 mg once daily; due to both the strong molecular interaction with the target and the wide volume of distribution, bodyweight does not affect the antitumoral activity of lenvatinib and the approved dose can be used without adjusting for weight. For the sake of completeness, a retrospective study on 70 patients with endometrial carcinoma treated with the combination of lenvatinib and pembrolizumab showed that a lower starting dose of lenvatinib (14 mg daily) was as similarly effective and safe than the full dose, with a significantly lower prevalence of dose reductions (15). Further trials may better elucidate this point.

Data from the real world would be also helpful to further improve the management of endometrial cancer with lenvatinib. Up to date, few data are available: a Korean multicenter study described similar activity and discontinuation rates as clinical trials. Patients received the combination of lenvatinib and pembrolizumab for a median of 4.5 cycles, achieving the best objective response rate and disease control rate of 23.8% (95% CI, 11.9–38.1) and 76.2% (95% CI, 61.9–88.1), respectively. Overall, 56.2% of patients needed lenvatinib dose reduction once or more (16).

## Lenvatinib plus pembrolizumab activity on endometrial cancer: Results from KEYNOTE 146 and KEYNOTE 775 trials

Recent advances in immunotherapy demonstrated the efficacy of pembrolizumab in solid tumors with MSI-H, dMMR, or with a high tumor mutational burden (4). Adding lenvatinib to an immune checkpoint inhibitor determined the synergic effect, described above, which provided a rationale for clinical trials.

KEYNOTE 146, a phase Ib/II trial, investigated lenvatinib plus pembrolizumab beyond first-line treatment in selected advanced solid tumors, including endometrial cancer, and established the recommended phase II dose to be lenvatinib 20 mg orally daily with pembrolizumab 200 mg intravenously every 3 weeks. This trial enrolled patients with metastatic endometrial cancer, unselected for microsatellite instability or PD-L1, thus including those patients who were less or not responsive to immunotherapy (5, 6). Overall, 38.0% of patients achieved the objective response at 24 weeks and most patients showed a reduction in tumor size, although not sufficient to define a complete or partial response according to RECIST 1.1 criteria (6). These results led to the accelerated approval of lenvatinib plus pembrolizumab by the Food and Drug Administration for the treatment of advanced endometrial carcinoma that is not MSI-H or dMMR, after progression with prior systemic therapy.

In the phase III trial KEYNOTE 775, the combination was compared with chemotherapy of investigator’s choice (doxorubicin or paclitaxel) beyond first-line treatment (17): in both patients with proficient-MMR (pMMR) and all-comers, lenvatinib plus pembrolizumab achieved a significantly longer progression-free survival (PFS), doubled overall response rate (ORR), and gained a longer duration of response. A *post hoc* analysis described the activity of lenvatinib plus pembrolizumab that was maintained in all histology subtypes, including serous and clear cell histology, which currently represent an unmet clinical need; prior therapy and platinum-free interval did not affect PFS (18).

In line with PFS data, the overall survival (OS) improved in all-comers and pMMR (17) and this result was consistent in all subgroups and histologic subtypes (18). The effect on OS was not influenced by one prior platinum-based treatment, but more than one previous platinum-based treatment reduced the response, thus suggesting that this treatment might be used early in the therapeutic strategy; the efficacy was maintained independently of platinum-free interval.

In November 2021, European Medicine Agency has approved lenvatinib plus pembrolizumab for the treatment of advanced or recurrent endometrial carcinoma in adults who have disease progression on or following prior treatment with a platinum-containing therapy in any setting and who are not candidates for curative surgery or radiation.

## Safety data

### Lesson from clinical trials

Concerning safety, in the setting of endometrial cancer, few institutes and clinicians have already gained experience with lenvatinib plus pembrolizumab; therefore, it is important to take into account the experience of pivotal trials and other diseases where the combination is already used in clinical practice. In addition, it should be considered that endometrial cancer prevalently affects elderly women with multiple comorbidities that can exacerbate adverse events potentially related to this treatment.

In KEYNOTE 775 trial, all patients experienced at least one adverse event related to therapy; 66.5% of patients required a dose reduction after adverse events, 33.0% discontinued the treatment, and 69.2% had a transient interruption to manage toxicities. The most frequently reported adverse events were: hypertension, an on-target effect, hypothyroidism, diarrhea, nausea, vomiting, loss of appetite, fatigue, and weight loss (17).

**Figure 1:**
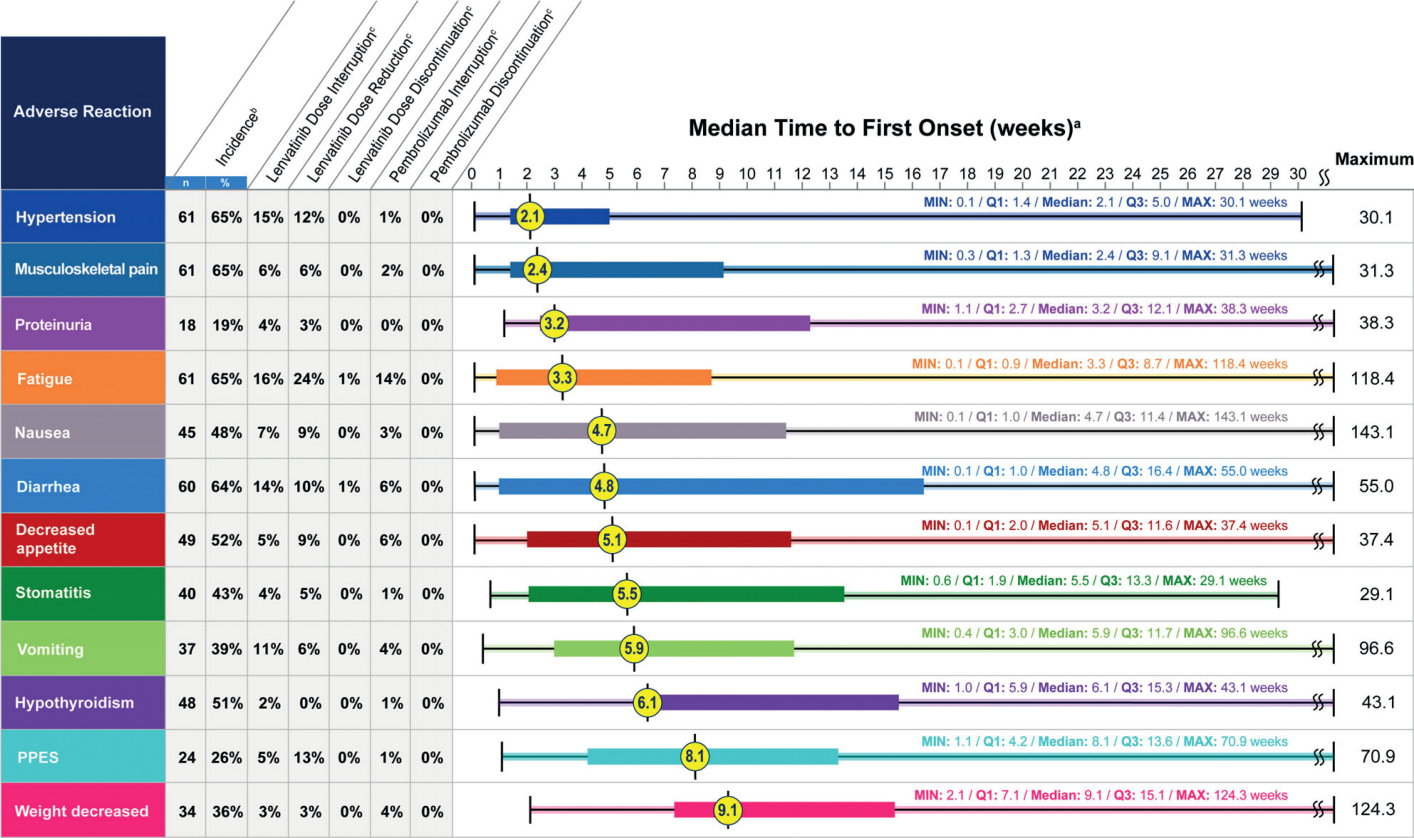
*Post hoc* analysis of time to first onset of selected adverse reactions. Reproduced with permission from (19).

Toxicities are not related to subgroup populations, as the pMMR population showed a similar safety profile as all-comer. When adjusted for exposure, the most frequent adverse events were diarrhea, hypertension, and musculoskeletal disorders in both all-comer and pMMR populations. During the trial, these toxicities had been managed with dose reductions and interruptions of lenvatinib without observing a decreased activity of drugs. Indeed, despite dose reductions, median tumor size decreased over time (18). The median time to the first onset of most frequent adverse events occurred approximately 3 months after treatment initiation in both all-comer and pMMR populations: adverse events with the shortest median time to onset included hypertension and musculoskeletal disorders, while hypothyroidism, palmar-plantar erythrodysesthesia (PPES), and weight decrease, which can be considered as a cumulative effect of vomiting, nausea, and musculoskeletal disorders, had a long time to onset (**Figure 1**) (19). Therefore, some adverse events should be monitored and prevented from the beginning of the treatment. Furthermore, being proactive in the management of gastrointestinal adverse events may avoid weight loss. Patients’ and clinicians’ education and preventive strategies need to be implemented (18, 19).

Health-related quality of life (HRQoL) assessment in the KEYNOTE 775 revealed a similar profile between lenvatinib plus pembrolizumab and the chemotherapy of choice, thus supporting the favorable risk/benefit ratio of the combination (20).

### Lesson from thyroid carcinoma and hepatocellular carcinoma

The lesson learned from thyroid carcinoma and hepatocellular carcinoma (HCC) might be useful to manage adverse events even in patients with endometrial carcinoma. In patients with radioiodine (RAI) refractory differentiated thyroid carcinoma, during the SELECT trial, which compared lenvatinib 24 mg to placebo, the most frequent adverse events included hypertension (68%), diarrhea (59%), fatigue (59%), stomatitis (36%), PPES (32%), and proteinuria (31%) (21). Similar adverse events were reported in clinical practice, with the most common being fatigue (13.6%) and hypertension (11.6%) (22). Hypertension, fatigue, and diarrhea appear relatively early during lenvatinib treatment. They can be managed with appropriate drugs, considering progressive dose reduction in case of ineffectiveness. Symptomatic biliary disorders (gallbladder and biliary duct disease) and cholecystitis (generally acalculous) were reported after 4 months of lenvatinib initiation or even later. The onset of symptoms and the peak of γ-glutaryl transferase levels corresponded to the highest weight loss during the first months of treatment. When these disorders required surgical intervention, presurgical lenvatinib interruption was shorter than one week (at least 48 hours before) and the treatment was resumed immediately after wound healing (23); in other cases, supportive care with ursodeoxycholic acid, when appropriate, and TKI dose reduction were used (24). Few cases of fistula and tumor-related bleeding were described after 10 weeks of treatment with lenvatinib; individual dose adjustments should be considered to manage this adverse reaction (25). Hemorrhage, acute coronary syndrome, and thrombosis/venous thromboembolism also occurred in clinical practice and should be kept into account in the management of lenvatinib (26). The ability to manage toxicities determined longer treatment duration and allowed to observation of late adverse events that could not be found in clinical trials. A retrospective study revealed new adverse events after 12 months of lenvatinib treatment: cardiovascular toxicity was the most common (57%) and no differences in the incidence of late adverse events were observed between younger (<65 years) and older patients (≥65 years), except for QTc prolongation that was more frequent in older people (27).

Patients with hepatocellular carcinoma frequently present a liver disease in addition to cancer. From a phase I trial, lenvatinib 12 mg once daily was determined to be the dose that achieved preliminary efficacy with manageable toxicity and was the recommended dose for patients with HCC with liver function as Child Pugh‐A, while for patients with Child Pugh-B the recommended dose was 8 mg (28–30). Since weight loss determined an increase in the area under curve, dose adjustment according to bodyweight was suggested (31). In particular, in patients with HCC Child-Pugh class A starting doses of 12-mg and 8-mg for subjects over 60 kg and under 60 kg of body weight respectively, were used in the phase 3 REFLECT trial and are now the recommended doses for patients with HCC. The main toxicities that emerged from the phase III trial were hypertension (42.2%), diarrhea (38.7%), decreased appetite (34.0%), and weight loss (30.9%); PPES was reported by 26.9% of patients versus 52.4% in the group randomized to receive sorafenib (32). In clinical practice, similar adverse events were reported with a high incidence of hypertension, diarrhea, and anorexia/weight loss (33). Monitoring blood pressure and body weight from treatment initiation and educating the patient and his/her caregiver to promptly recognize any problem allow to manage these adverse events as well.

The combination of lenvatinib and pembrolizumab was tested also in other solid tumors - melanoma, renal cell carcinoma, squamous cell carcinoma of the head and neck, non-small cell lung cancer, or urothelial cancer- in a phase Ib/II open-label study in which all patients received the recommended phase II dose of lenvatinib 20 mg/day with pembrolizumab 200 mg every 3 weeks until disease progression or development of unacceptable toxicity. The combination confirmed a manageable safety profile in patients with these solid tumor types, with fatigue, hypertension, diarrhea, and hypothyroidism being the most common adverse reactions (34).

Even in patients with renal cell carcinoma enrolled in the CLEAR trial, lenvatinib plus pembrolizumab demonstrated a safety profile consistent with that previously described for each drug both as a single agent and combined. Adverse reactions that occurred or worsened during treatment led to a dose reduction of lenvatinib in 68.8% of patients treated with the combination. Again, interruptions and reductions were effectively utilized in the study, which allowed patients to continue to receive life-prolonging therapy for a longer period (35). The analysis of HRQoL data demonstrated that lenvatinib plus pembrolizumab had similar or favorable scores compared with those obtained by sunitinib, especially concerning the time to the definitive deterioration (36).

## Lenvatinib-related adverse events: Incidence, time to the first onset, and suggestions for an appropriate management

### Hypertension

Hypertension is one of the adverse events most frequently reported with lenvatinib and other anti-angiogenic; it is an on-target adverse event, directly related to the activity of these drugs on their target. Indeed, the inhibition of the VEGFR signaling pathway acts on nitric oxide-dependent processes and impairs endothelium-dependent vasodilation in the microvessels, as well as seems to enhance the vasomotor tone through the endothelin system with a mechanism that has not been elucidated yet (37). Regardless of specific angiogenic inhibitors, most patients experience an increase in blood pressure with a peak within the first weeks of treatment (37).

In KEYNOTE 146 trial, hypertension occurred in approximately 65% of patients treated with lenvatinib in the first two weeks of treatment (19). Prevention, early detection, and effective management of hypertension are important to minimize the need for dose interruptions and reductions. Before starting lenvatinib, blood pressure should be measured and eventually controlled with a stable antihypertensive therapy. Proactive management includes prompt, daily monitoring of blood pressure before the start of lenvatinib and from the first cycle both at the clinic visit and at home. Administration of angiotensin-converting enzyme inhibitors, angiotensin receptor blockers, or beta-blockers is useful in the occurrence of a hypertensive peak; calcium channel blockers should be used with caution to avoid drug-drug interactions, which, however, are limited with lenvatinib (38–40). The choice of antihypertensive treatment should be individualized to the patient’s clinical characteristics and follow standard medical practice: for previously normotensive subjects, monotherapy with one of the classes of antihypertensives should be started when elevated blood pressure is observed, while for patients already on hypertension treatment, increasing the dose of the current agent or adding a different class of antihypertensive should be appropriate (40). If antihypertensive agents are not effective or hypertension grade 3 occurs, lenvatinib dose reduction may be necessary (39).

Patients should be educated to reduce the risk factors that can enhance hypertension, such as smoking, alcohol consumption, stress, and lack of physical activity.

### Musculoskeletal pain

Musculoskeletal pain was experienced in 65% of patients in the first 2.4 weeks of treatment (19). This adverse event should be managed with medications, including opioids (oxycodone, fentanyl, morphine), paracetamol, nonsteroidal anti-inflammatory drugs, and topical diclofenac (6).

### Fatigue

In the clinical trial, fatigue was reported in 65% of patients, with a median time to the first onset of 3.3 weeks (19). Managing fatigue can be difficult; the initial step is to exclude any treatable cause that presents similar effects (e.g anemia) and assess the intensity level of fatigue if present. Then, periodic re-evaluations are recommended at routine and follow-up visits (41). Based on the experience in hepatocellular carcinoma, Grade 1 fatigue can generally be managed without interrupting lenvatinib. If fatigue of Grade≥2 occurs in the early phase of treatment, lenvatinib should be resumed at a reduced dose after fatigue is resolved, while it can be restored at a full dose if fatigue occurs after several weeks of treatment. Only if a patient cannot tolerate the symptoms of Grade 2 fatigue, lenvatinib should be discontinued (42). Education (i.e. coping strategies and good sleep hygiene), counseling, and both non-pharmacologic (physical activity, nutritious diet, and proper hydration) and pharmacologic interventions may be introduced to ameliorate fatigue, accounting that in many instances a combination of approaches must be used (39, 41). Lenvatinib dosing in the evening rather than in the morning may reduce daytime fatigue (39).

### Stomatitis

Stomatitis or mucositis is frequently reported in patients receiving TKI and other targeted therapies. Stomatitis is a painful inflammation of the mucous lining of the mouth, whereas mucositis refers to inflammation or ulceration of the mucous membranes lining the digestive tract; both adverse events can make it difficult to speak, eat, or even open the mouth and generate discomfort for the patient. In KEYNOTE 146 trial, stomatitis occurred in 43% of patients after a median of 5.5 weeks (19); only 5% of patients required a reduction of lenvatinib for this adverse event, while 25.5% of patients received symptomatic medications reported to manage this adverse event, such as dexamethasone, lidocaine, triamcinolone, nystatin, and mouth preparations (6). To minimize the risk of stomatitis and improve adherence to therapy, patient awareness and early intervention are important. Patients should be educated to avoid mint-flavored toothpaste, alcohol-containing mouthwash, and spicy or acidic foods and to maintain dental and oral care. Furthermore, before lenvatinib initiation and regularly during the treatment, accurate oral health is recommended. Topical lidocaine or steroid ointment may also be helpful for painful ulcerations, although for more severe stomatitis (grade ≥3) dose reductions or interruptions may be necessary (43).

### Diarrhea

In KEYNOTE 146, 64% of patients experienced diarrhea, with a median time to the first onset of 4.8 weeks (19). Recommendations for lenvatinib-associated diarrhea management are consistent across tumor types. Diarrhea may be managed by making dietary changes, including avoiding caffeine, alcohol, spicy or fatty foods, dairy products, and foods high in insoluble fibers; writing a food diary may help identify particular items that exacerbate diarrhea. Patients with diarrhea should not become dehydrated; therefore, fluid intake should be increased, and electrolytes monitored and replaced when necessary. Prompt medical management of diarrhea should be established to prevent dehydration before any lenvatinib therapy dose interruption or reduction. When pharmacological intervention is necessary, loperamide is widely recommended; atropine-diphenoxylate, octreotide, codeine, or tincture of opium can be also prescribed (39). In case of Grade 3 diarrhea, lenvatinib should be interrupted until resolution, and resumed at a reduced dose; if diarrhea is persistent and becomes Grade 4 despite medical management, lenvatinib should be discontinued. Patients should be encouraged to complete a stool diary and report any concerns to their healthcare provider. Data from the clinical trials indicate that no patients discontinued pembrolizumab because of diarrhea and among patients who reported an adverse event 6% required pembrolizumab dose interruption, compared to 14% who needed lenvatinib dose interruption (19). Prescribing information of pembrolizumab should be considered to eventually interrupt or discontinue the drug in presence of diarrhea of grade 3 and 4 (or recurrent grade 3), respectively (44).

### Nausea/vomiting

Nausea and vomiting were reported in 48% and 39% of patients, respectively (19). As for other TKIs, most cases of nausea and vomiting are of Grade 1 or 2. Optimal medical management to minimize gastrointestinal toxicity should be initiated before any lenvatinib interruption or dose reduction. Dietary modifications (i.e. avoiding chocolate, caffeine, alcohol, and nicotine) are suggested to prevent this adverse event, and antiemetics may alleviate symptoms. Caution should be paid in prescribing ondansetron as it may determine QTc prolongation (39).

### Weight loss and anorexia

Decreased appetite was reported in 52% of patients, with a median time to onset of 5.1 weeks, while weight loss occurred in 36% of patients and had a late-onset after 9.1 weeks from starting the combination (19). Prophylactic recommendations for decreased appetite and weight loss include monitoring the patient’s appetite and weight in each treatment cycle and encouraging a nutritious diet. In the case of Grade 1 and 2, anorexia dose interruption is effective to alleviate symptoms and lenvatinib treatment can be resumed at the same dose; in the case of Grade 3 anorexia occurring several days after lenvatinib initiation, dose interruption, and dose reduction should be considered (42). Antiemetic agents (prochlorperazine maleate or domperidone) can be prescribed, or oral nutrition support offered when underlying nausea is present (39).

### Proteinuria

Proteinuria occurred in 19% of patients enrolled in the KEYNOTE 146 trial in the first 3 weeks (19). Before starting lenvatinib, it is mandatory to check renal function for the presence of proteinuria, and during the treatment monitoring patients with urine dipstick testing regularly is recommended (40). Special attention should be paid to patients with renal dysfunction caused by diabetes or hypertension during lenvatinib treatment. If Grade 1 in high-risk patients with edema, fluid collection, or elevated serum creatinine, lenvatinib treatment should be interrupted and spot urine or 24-hr urine should be checked to determine urinary protein and/or the urine protein-to-creatinine ratio. In the case of Grade 2 proteinuria, dose interruptions, adjustments, or discontinuation may be required (40).

### Thyroid toxicity

Hypothyroidism was the most common thyroid toxicity described in the KEYNOTE 146 trial in 51% of patients; the median time to the first onset was 6.1 weeks (18). The American Thyroid Association 2015 guidelines for adult patients recommend monitoring thyroid function by testing thyroid-stimulating hormone (TSH) levels at baseline and regularly during treatment (39). Because patients with Grade 2 hypothyroidism tend to have no symptoms, patients requiring levothyroxine can be hard to identify; however, appropriate information on how to manage hypothyroidism must be provided to clinicians (37). According to prescribing information for both lenvatinib and pembrolizumab, hypothyroidism either immuno-related or associated with lenvatinib should be treated per clinical practice with a substitutive therapy (44, 45).

### PPES

PPES was reported in 26% of patients, with a median time to the first onset of 8.1 weeks (19); 11.1% of patients received medications, including emollients and protectants, and corticosteroids (6). Physicians and nurses should educate patients to care for the skin on their hands and feet before lenvatinib initiation, highlighting the importance of moisturizing hands and feet, the use of appropriate protective clothing, and the importance of sun protection. A change in the

schedule of lenvatinib treatment should be considered according to the severity of PPES. In the case of Grade 1 PPES, lenvatinib treatment may be continued at the same dose with the use of moisturizing cream, and a hydrocolloid dressing for the feet may be considered; to manage Grade 2-3 PPES lenvatinib should be interrupted and steroid ointment should be used, after consulting a dermatologist. After recovery to a lower grade, lenvatinib treatment may be resumed at a reduced dose (40).

### Alterations in cardiac function

In KEYNOTE 146 10% of patients reported QTc prolongation and cardiac dysfunction (6). The incidence was similar to that observed in the SELECT trial in radioiodine refractory patients where 9% of patients (2% had a cardiac adverse event of Grade 3) experienced cardiac dysfunction, including decreased left or right ventricular function, cardiac failure, or pulmonary edema (21). The increased risk of hypertension associated with lenvatinib may also determine an increased risk of cardiac disease. A baseline echocardiogram is recommended before starting lenvatinib and regularly during the treatment (at least once a year); administration of heart failure therapies is also recommended if indicated (43). Lenvatinib interruption should be considered for grade 3 cardiac dysfunction until resolution to grade 0 or 1. Upon resolution, lenvatinib can be resumed at a lower dose and blood pressure should be monitored daily and maintained within the normal range. Lenvatinib discontinuation should be considered for grade 4 cardiac dysfunction (43).

### Osteonecrosis

Osteonecrosis is a rare adverse event associated with antiangiogenic agents, even if its incidence is unknown because it is not always monitored. Before starting lenvatinib, ortho-pan tomography and dental visit are suggested, and invasive dental procedures should be avoided during treatment.

## Discussion

In clinical trials, the combination of lenvatinib plus pembrolizumab obtained a significant clinical benefit in terms of PFS and OS in patients with endometrial carcinoma. The safety profile was consistent with the established profiles of each drug in monotherapy. Immune-related adverse events reported in the study, including colitis, rash, hepatitis, and pneumonitis, are expected to occur with anti-PD-1 therapy, likely because of general immunologic enhancement. No new safety signals were identified, and the toxicity profile was manageable with supportive medications, dose interruptions, and/or lenvatinib dose reductions (19). Therefore, to translate advantages observed in trials to clinical practice and provide the maximal benefit from the treatment to patients, clinicians should learn how to manage lenvatinib and its potentially related adverse events.

An appropriate, proactive, and thorough management of lenvatinib toxicities is required to maximize lenvatinib efficacy. Adverse events should be detected as early as possible, by both carefully monitoring the patient from lenvatinib initiation and preventing their occurrence. Patients should be carefully followed also during treatment as some adverse events, e.g. cardiac dysfunction may appear later. Increased awareness on risk to benefit ratio among clinicians would also be helpful to avoid dose interruptions or discontinuation in case of adverse events, with preferring other medical interventions and supportive care, as the experience in thyroid carcinoma and hepatocellular carcinoma teaches (46).

Indeed, evidence in the setting of RAI-refractory differentiated thyroid carcinoma indicated that dose interruptions of more than 10% correlated to shorter PFS, thus limiting the clinical benefit of the drug (47). Furthermore, starting at a reduced dose did not show any advantage in the incidence of adverse events of grade 3, but provided a clinically relevant difference in terms of a lower overall response rate (14). Therefore, dose reductions are justified to manage adverse events, but not to prevent them.

Patients and their caregivers should be educated and made aware of the importance of adherence to treatment to optimize its effectiveness. On one hand, patients should be educated to reduce the risk factors associated with adverse events: smoking, alcohol consumption, stress, and lack of physical activity should be avoided to prevent hypertension, as well as nutrition counseling can be useful to reduce gastrointestinal disorders and weight loss. On the other, increasing patients’ awareness of signs and symptoms of a potential adverse event can result in early detection and appropriate management before an excessive worsening. Effective communication between patients and their physicians is a key factor in successful long-term treatment with lenvatinib.

To date, the experience of handling lenvatinib in endometrial carcinoma is very limited in clinical practice, and in this setting, patients are usually elderly with concomitant diseases that lenvatinib may exacerbate; a geriatric assessment is recommended upfront to effectively plan a monitoring activity and more tailored supports. A long-term follow-up may reveal further adverse events that clinical trials could not detect due to the short duration of treatment. Future data collection in the real world also in patients with endometrial carcinoma will allow to better address the management of adverse events and maximize the clinical benefit for all patients.

## Conclusion

The toxicity profile associated with lenvatinib in endometrial cancer is similar to that reported in thyroid carcinoma and hepatocellular carcinoma, where lenvatinib is already used in clinical practice. Careful management of adverse events with prevention strategies, early detection, and proactive interventions allows patients to remain on full doses of lenvatinib as long as possible to gain maximal benefit from the treatment.

## Author contributions

Each author gave a substantial contribution to designing the work and collecting data, revised critically for important intellectual content, approved the final version, and agreed to be accountable for all aspects of the work.

## Acknowledgments

The authors thank Content Ed Net for editorial support, with the helpful contribution of medical writer Elisa Sala, Ph.D.

## Conflict of interest

UDG has been consultant or advisory to Astellas, AstraZeneca, Bayer, BMS, Clovis, Eisai, Ipsen, Janssen, MSD, Novartis, Pfizer, PharmaMar, Roche, and received institutional research grants from AstraZeneca, Sanofi and Roche. GV has been consultant for Eisai. NC received grants or contracts from AstraZeneca, PharmaMar and Roche, payment or honoraria for lectures, presentations, speakers bureaus, manuscript writing or educational events from AstraZeneca, Tesaro, Novartis, Clovis Oncology, Merck Sharp and Dohme, GlaxoSmithKline and Eisai, participated in a Data Safety Monitoring Board or Advisory Board for Roche, PharmaMar, AstraZeneca, Clovis Oncology, Merck Sharp and Dohme, GlaxoSmithKline, Tesaro, Pfizer, BioCad, Immunogen, Mersana, Eisai, Oncxema and Nuvation Bio, has been leadership or fiduciary role in the steering

Committee member on ESMO clinical guidelines and a Scientific Committee Chair for Acto Onlus. SP received honoraria from Astrazeneca, MSD, Roche, Pfized, GSK, Clovis Oncology, research funding from Astrazeneca, MSD, Roche, Pfized, GSK and has been consultant for Eisai. LL received conference honoraria/Advisory Board from EISAI, MSD, Merck Serono, McCann Healthcare, Eli Lilly, Sanofi, Sunpharma, IPSEN, Bayer and has been consultant for Eisai. AG has been consultant for Eisai. RD has been consultant, speakers bureau, advisory board member for Ipsen, Novartis, Pfizer, Sanofi Genzyme, AstraZeneca, Janssen, Gilead, Lilly, Gilead, GSK, EUSA Pharma and EISAI. DL participated in the advisory board of GSK, AstraZeneca, MSD, Clovis Oncology, Pharmamar, Merck Serono, Seagen, Immunogen, Genmab, Oncoinvest, Corcept, Sutro. has been consultant for Pharmamar, Amgen, AstraZeneca, Clovis Oncology, GSK, MSD, Immunogen, Genmab, Seagen, EISAI, received institutional funding from MSD, Clovis Oncology, GSK, Pharmamar, AstraZeneca, Seagen, Genmab, Immogen, Incyte, Novartis, Roche, has been invited Speaker for Genmab, Pharmamar, MSD and Principal Investigator for Astra Zeneca, MSD, Genmab, Immunogen, Clovis, Roche, Incyte, and board of Directors for GCIG. SR received conference and advisory board honoraria from EISAI, GSK, MSD, Pfizer, Janssen, Astellas, Ipsen, BMS, Clovis and has been consultant for Eisai. GM has been consultant for Eisai. AS has been consultant for Eisai. CZ has been consultant for Eisai.

## Publisher’s note

All claims expressed in this article are solely those of the authors and do not necessarily represent those of their affiliated organizations, or those of the publisher, the editors and the reviewers. Any product that may be evaluated in this article, or claim that may be made by its manufacturer, is not guaranteed or endorsed by the publisher.
