# Corrigendum: Optimizing the use of lenvatinib in combination with pembrolizumab in patients with advanced endometrial carcinoma

**DOI:** 10.3389/fonc.2023.1232476

**Published:** 2023-08-07

**Authors:** Domenica Lorusso, Romano Danesi, Laura Deborah Locati, Gianluca Masi, Ugo De Giorgi, Angiolo Gadducci, Sandro Pignata, Roberto Sabbatini, Antonella Savarese, Giorgio Valabrega, Claudio Zamagni, Nicoletta Colombo

**Affiliations:** ^1^Department of Clinical Research Planning, Fondazione Policlinico Universitario A Gemelli Istituto di Ricerca e Cura a carattere scientifico (IRCCS), Rome, Italy; ^2^Department of Life Science and Public Health, Catholic University of Sacred Heart, Rome, Italy; ^3^Unit of Clinical Pharmacology and Pharmacogenetics, Department of Clinical and Experimental Medicine, University of Pisa, Pisa, Italy; ^4^Translational Oncology Unit, Istituto di Ricerca e Cura a carattere scientifico (IRCCS) Istituti Clinici Scientifici (ICS) Maugeri, Pavia, Italy; ^5^Department of Internal Medicine and Medical Therapy, University of Pavia, Pavia, Italy; ^6^Unit of Medical Oncology 2, Azienda Ospedaliero-Universitaria Pisana, Pisa, Italy; ^7^Department of Translational Research and New Technologies in Medicine and Surgery, University of Pisa, Pisa, Italy; ^8^Department of Medical Oncology, Istituto di Ricerca e Cura a carattere scientifico (IRCCS) Istituto Romagnolo per lo Studio dei Tumori (IRST), Dino Amadori, Meldola, Italy; ^9^Department of Clinical and Experimental Medicine, Division of Gynecology and Obstetrics, University of Pisa, Pisa, Italy; ^10^Department of Urology and Gynecology, Istituto Nazionale Tumori Istituto di Ricerca e Cura a carattere scientifico (IRCCS) “Fondazione Giovanni Pascale”, Naples, Italy; ^11^Division of Medical Oncology 1, Istituto di Ricerca e Cura a carattere scientifico (IRCCS) -Regina Elena National Cancer Institute, Rome, Italy; ^12^University of Torino-Struttura Complessa a Direzione Universitaria (S.C.D.U.) Oncologia Azienda Ospedaliera (A.O) Ordine Mauriziano-Ospedale Umberto I, Torino, Italy; ^13^Addarii Medical Oncology, Istituto di Ricerca e Cura a carattere scientifico (IRCCS) Azienda Ospedaliero-Universitaria di Bologna, Bologna, Italy; ^14^School of Medicine and Surgery, University of Milan-Bicocca, Milan, Italy; ^15^Department of Oncological Gynecology, European Institute of Oncology (IEO) Istituto di Ricerca e Cura a carattere scientifico (IRCCS), Milan, Italy

**Keywords:** lenvatinib, pembrolizumab, endometrial cancer, tyrosine kinase inhibitor, immune response

In the published article, there was an error in **Figure 1** as published. By mistake, we added the reference 18 for this figure. The corrected **Figure 1** and its caption appear below.

In the published article, an author name was incorrectly written as Sabbatini Roberto. The correct name is Roberto Sabbatini.

The authors apologize for these errors and state that these do not change the scientific conclusions of the article in any way. The original article has been updated.

## Figures and Tables

**Figure 1:**
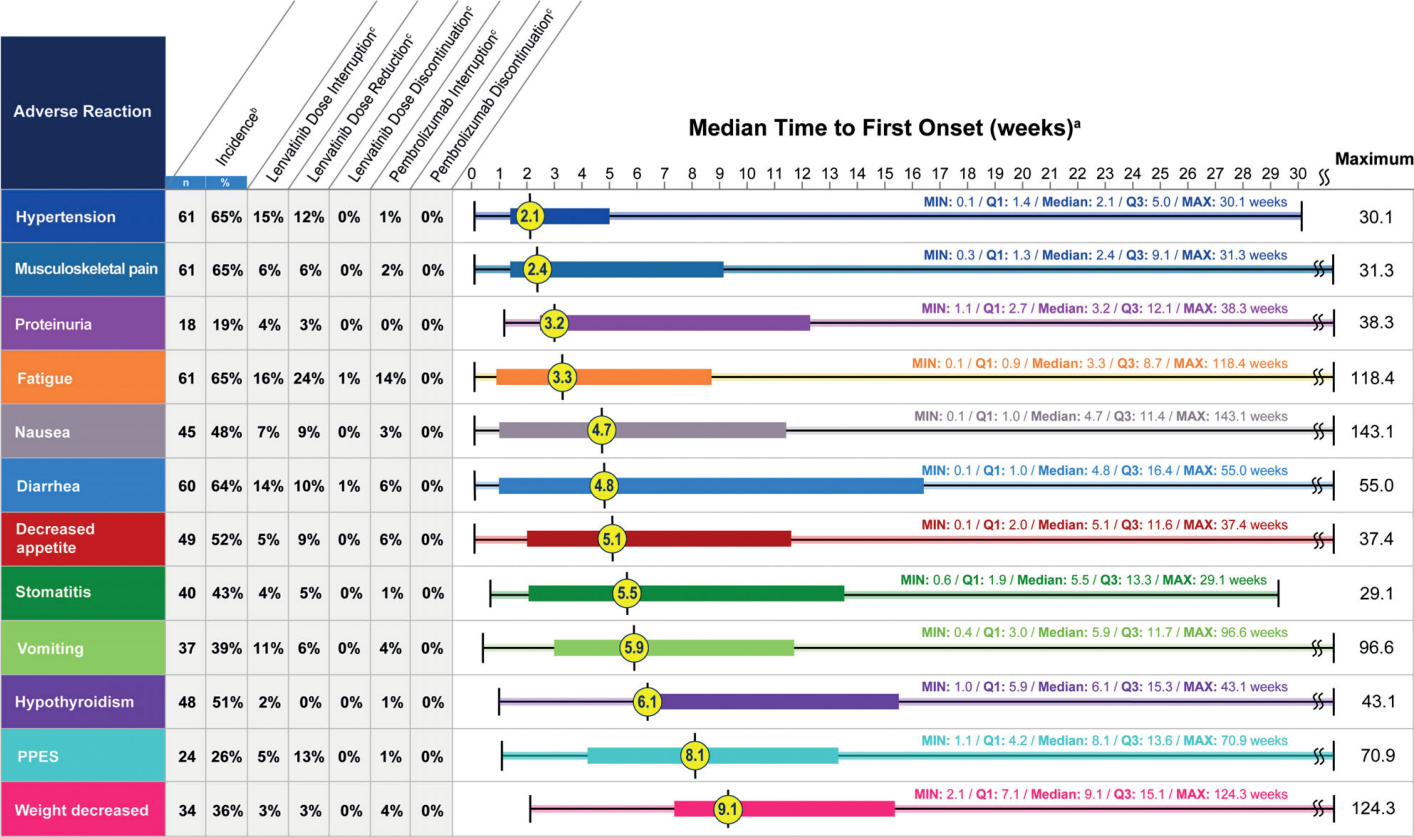
*Post hoc* analysis of time to first onset of selected adverse reactions. Reproduced with permission from [19].

